# A comparison of small-area deprivation indicators for public-health surveillance in Sweden

**DOI:** 10.1177/14034948211030353

**Published:** 2021-07-20

**Authors:** Ulf Strömberg, Amir Baigi, Anders Holmén, Brandon L. Parkes, Carl Bonander, Frédéric B. Piel

**Affiliations:** 1 School of Public Health and Community Medicine, Institute of Medicine, Sahlgrenska Academy at University of Gothenburg, Sweden; 2 Department of Research and Development, Region Halland, Sweden; 3 UK Small Area Health Statistics Unit (SAHSU), Department of Epidemiology and Biostatistics, School of Public Health, Imperial College London, UK; 4 MRC Centre for Environment and Health, School of Public Health, Imperial College London, UK; 5 National Institute for Health Research Health Protection Research Unit (NIHR HPRU) in Environmental Exposures and Health, Imperial College London, UK

**Keywords:** Factor analysis, mortality, spatial smoothing, small-area characteristics, sociodemographic factors, Sweden

## Abstract

**Aims:**

The aims of this study were to construct a small-area index of multiple deprivation (IMD) from single deprivation indicators (SDIs) and to compare the explanatory power of the IMD and SDIs with regard to mortality. We considered a small-area division of Sweden consisting of 5985 DeSO (*Demografiska statistikområden*), each with a population size between 653 and 4243 at the end of 2018.

**Methods:**

Four SDIs were provided by open-source data: (a) the proportion of inhabitants with a low economic standard; (b) the proportion of inhabitants aged 25–64 years with ⩽12 years of schooling; (c) the proportion of inhabitants aged 16–64 years who were not in paid employment; and (d) the proportion of inhabitants who lived in a rented apartment/house. A four-indicator IMD was constructed using factor analysis. As a validation, the IMD and SDIs were compared by exploring their DeSO-level associations with spatially smoothed death rates, with robustness checks of associations across different small-area contexts defined by degree of urbanisation and distribution of immigrants from non-Western countries.

**Results:**

The constructed IMD and SDI1 performed essentially equally and outperformed SDI2, SDI3 and SDI4. Associations between IMD/SDI1 and the spatially smoothed death rates were most pronounced within the age range 60–79 years, showing 5–8% lowered rates among those categorised in the least deprived quintiles of IMD and SDI1, respectively, and 7–9% elevated rates among those categorised in the most deprived quintiles. These associations were consistent within each small-area context.

**Conclusions:**

**We suggest prioritisation of SDI1, that is, a DeSO-level deprivation indicator based on open-access data on economic standard, for public-health surveillance in Sweden.**

## Introduction

Indicators of small-area deprivation and corresponding indices of multiple deprivation (IMD) can represent useful proxies covering various domains, such as income, education, employment and housing [1]. Single deprivation indicators (SDI) and IMDs are particularly useful when individual-level data are not easily available, which may be the case for regional authorities outside research institutions for example [2]. SDIs/IMDs should be kept distinct from data on health outcomes and preferably also from data on ethnicity/immigrant groups because the impact of ethnic composition or geographical origins of local population on health could be different from that of material deprivation [1,3].

IMDs have predominantly been developed at a national level to reflect within-country heterogeneities. They are commonly used in the UK [4–8] and have also been created for several other countries [2,9–12]. A Swedish index based on a geographical division referred to as SAMS (Small Areas for Market Statistics) was constructed for a study of neighbourhood deprivation influence on adolescent violent criminality and substance misuse, but only for SAMS in the three largest cities that had at least 500 inhabitants [13]. In 2018, the SAMS index was replaced by a new geographic division referred to as DeSO (*Demografiska statistikområden*). The DeSO geography was launched with the aim of facilitating the monitoring of segregation and socio-economic conditions in small geographic areas. The boundaries were defined with consideration of population size [14]. At the end of 2018, the population sizes across the 5985 DeSO varied between 653 and 4243.

### Aims

Our aim was twofold: (a) to construct, for the DeSO geography of Sweden, an IMD from four SDIs provided by open-source data, and (b) to compare the explanatory power of the SDIs and the constructed IMD with regard to mortality in order to prioritise deprivation measures to be considered for public-health surveillance in Sweden.

## Methods

### SDIs and IMD construction

From Statistics Sweden’s open-source database [15], we extracted DeSO-level data from the year 2018 on four SDIs : SDI1 – the proportion of inhabitants with a low economic standard (i.e. belonging to a household with a disposable income per consumption unit in the lowest quartile of all households in Sweden); SDI2 – the proportion of inhabitants aged 25–64 years with ⩽12 years of schooling; SDI3 – the proportion of inhabitants aged 16–64 years not in paid employment; and SDI4 – the proportion of inhabitants who live in a rented apartment/house. By combining these SDIs, we constructed an IMD through a factor analysis (considering the Varimax rotation method) to obtain corresponding latent variable of deprivation. We assigned the number of inhabitants as a weight to each DeSO, implying that each of the total 10,216,249 inhabitants contributed with his/her DeSO-level deprivation indicators. Redundancy between indicators of deprivation was measured by Bartlett’s test of sphericity [16]. The Kaiser–Meyer–Olkin (KMO) test was performed to measure the adequacy of the sampling [17,18]. SDIs with an eigenvalue ⩾1.0 were retained for further analysis [19], and factor loadings ⩾0.30, together with a total explained variance >50%, were considered meaningful [20]. The internal reliability was measured by Cronbach’s alpha coefficient [21].

### Exploring explanatory power

We explored associations between each SDI/IMD and the death rates for the year 2019. The number of deaths, stratified by DeSO, sex and age (five-year groups), were obtained from Statistics Sweden. The corresponding population data were extracted from Statistics Sweden’s open-source database [15]. We estimated spatially smoothed standardised mortality ratios (SMR) for each DeSO by using the Besag, York and Mollie spatial model [22] implemented in the Rapid Inquiry Facility 4.0, a disease mapping open-source application [23,24]. Let us use the notation ${\hat{\theta }}_{i}$ for a spatially smoothed SMR in DeSO *i* (*i* = 1,. . ., 5985). Associations on *ln*( ${\hat{\theta }}_{i}$ ) of each SDI_*i*_/IMD_*i*_, with and without accounting for context categories [1], were evaluated by ecological regressions (with inverse variances of *ln*( ${\hat{\theta }}_{i}$ ) incorporated as weights). Context categorisations were achieved by (a) the DeSO coding system, which makes distinction between rural, semi-urban or urban areas [25], and (b) ordering DeSO-level data on the proportion of non-western immigrants (i.e. inhabitants born in Eastern Europe, Asia, Africa or South America) from Statistics Sweden. These data were grouped into quintiles (Q1=lowest proportion, Q5=highest proportion).

## Results

A four-indicator IMD was formed as a single latent variable, with (a) an acceptable KMO value of 0.53; (b) *p*<0.0001 from Bartlett’s test of sphericity; (c) factor loadings 0.96, 0.24, 0.87 and 0.69 for SDI1, SDI2, SDI3 and SDI4, respectively; (d) 69% total explained variance; and (e) a satisfactory Cronbach’s alpha of 0.74. Table I presents descriptive statistics for this IMD, as well as each SDI. Segregation is more pronounced in urban areas, and hence neighbourhoods in Q1 (i.e. the least deprived quintile for an SDI/IMD) and Q5 (i.e. the most deprived quintile for an SDI/IMD) are relatively more frequent in urban than rural areas. Between 44% and 74% of the DeSO categorised in the most deprived quintiles did not match areas with the highest proportion of immigrants from non-Western countries.

**Table I.** Characteristics of four single indicators of small-area deprivation (SDI1, SDI2, SDI3 and SDI4) and a constructed index of multiple deprivation (four-indicator IMD) for Sweden.

	No. inhabitants in each DeSO in the end of year 2018, *M* (min–max)	SDI1: Proportion of inhabitants with low economic standard (median)	SDI2: Proportion of inhabitants aged 25–64 years with ⩽12 years of schooling (median)	SDI3: Proportion of inhabitants aged 16–64 years not in paid employment (median)	SDI4: Proportion of inhabitants living in a rented apartment/house (median)	No. DeSO categorised as rural/semi-urban/urban^a,b^	No. DeSO categorised in each quintile Q1/Q2/Q3/Q4/Q5 according to proportion of immigrants from non-Western countries^b^
*DeSO classified into quintiles of SDI1*:							
Q1	1741 (653–3275)	0.096	–	–	–	104/108/985	308/333/337/183/36
Q2	1702 (719–3565)	0.161	–	–	–	321/130/746	325/239/303/243/87
Q3	1631 (694–4243)	0.220	–	–	–	326/106/765	305/228/248/244/172
Q4	1650 (668–3204)	0.294	–	–	–	271/136/790	238/219/201/252/287
Q5	1789 (783–3419)	0.430	–	–	–	59/100/1038	81/132/160/215/609
*DeSO classified into quintiles of SDI2*:							
Q1	1754 (653–4243)	–	0.336	–	–	8/35/1154	160/262/429/256/90
Q2	1781 (719–3599)	–	0.505	–	–	65/104/1028	233/225/240/306/193
Q3	1725 (744–3207)	–	0.614	–	–	226/120/851	267/217/222/208/283
Q4	1654 (671–3034)	–	0.690	–	–	405/109/683	298/215/182/184/318
Q5	1620 (668–2931)	–	0.754	–	–	377/212/608	299/232/176/183/307
*DeSO classified into quintiles of SDI3*:							
Q1	1704 (706–3275)	–	–	0.111	–	289/128/780	478/322/249/131/17
Q2	1669 (694–3378)	–	–	0.145	–	338/131/728	314/271/317/222/73
Q3	1660 (653–3599)	–	–	0.176	–	278/117/802	262/242/284/273/136
Q4	1704 (725–4243)	–	–	0.223	–	145/117/935	147/203/244/310/293
Q5	1798 (783–3419)	–	–	0.328	–	31/87/1079	56/113/155/201/672
*DeSO classified into quintiles of SDI4*:							
Q1	1604 (706–2991)	–	–	–	0.013	429/61/707	430/258/230/143/136
Q2	1635 (653–2867)	–	–	–	0.065	526/139/532	403/319/222/170/83
Q3	1669 (668–3239)	–	–	–	0.196	122/235/822	245/248/325/232/147
Q4	1788 (789–4243)	–	–	–	0.386	3/120/1074	137/219/280/315/246
Q5	1839 (838–3599)	–	–	–	0.721	1/7/1189	42/107/192/277/579
*DeSO classified into quintiles of the 4-indicator IMD*:							
Q1	1746 (653–3275)	0.096	0.441	0.116	0.028	114/111/972	331/332/332/171/31
Q2	1671 (755–3239)	0.163	0.594	0.142	0.079	402/125/670	352/246/288/214/97
Q3	1617 (671–4243)	0.221	0.641	0.171	0.172	379/111/707	334/221/247/242/153
Q4	1675 (668–3599)	0.290	0.672	0.216	0.341	175/151/871	181/238/227/283/268
Q5	1825 (783–3419)	0.429	0.705	0.324	0.681	11/82/1104	59/114/155/227/642

Each SDI/IMD is divided into quintiles, each comprising 1197 small areas (DeSO), where Q1=the least deprived areas and Q5=the most deprived areas.

a No. inhabitants in rural DeSO in the end of 2018 (*M* (min–max)): 1467 (653–2691); in semi-urban DeSO: 1540 (694–2948); and in urban DeSO: 1789 (668–4243).

b Percentages in each category are not presented. Percentages are proportional to the numbers presented (adding up to 1197 in each deprivation quintile).

**Figure 1.**
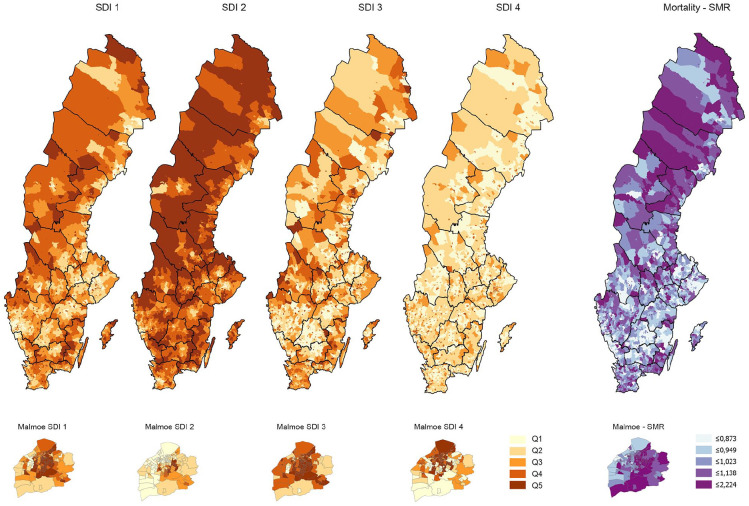
Maps visualising geographical variations for each single deprivation indicator: SDI1, proportion of inhabitants with a low economic standard (i.e. belonging to a household with a disposable income per consumption unit in the lowest quartile of all households in Sweden); SDI2, proportion of inhabitants aged 25–64 years with ⩽12 years of schooling; SDI3, proportion of inhabitants aged 16–64 years not in paid employment; and SDI4, proportion of inhabitants who live in a rented apartment/house, within the whole of Sweden (with the 21 regions marked) and, in enlarged maps, within the municipality of Malmö, to illustrate changing patterns in a predominately urban area (including several small-area DeSO with high population density). Analogous maps of the spatially smoothed standardised mortality ratios within the age span 60–79 years are also shown.

Figure 1 visualises the geographical distributions of each SDI and the spatially smoothed SMRs within the age span 60–79 years. Associations between SDIs/IMD and death rates within this age span were more pronounced than associations between SDIs/IMD and death rates in other age groups (cf. Table II and Supplemental Table SI). The constructed four-indicator IMD and SDI1 showed the best explanatory power, robustly over each context. Across the quintiles of the IMD (or SDI1), the crude death rate increased gradually from 5.2 (5.1) to 10.9 (10.6) per 1000 persons within the age group 60–69 years, and from 15.5 (15.5) to 26.9 (27.4) per 1000 within the age group 70–79 years (Table II). Associations between IMD/SDI1 and the spatially smoothed death rates within the age span 60–79 years showed 5–8% lowered rates among those categorised in the least deprived quintiles of IMD and SDI1, respectively, and 7–9% elevated rates among those categorised in the most deprived quintiles. These gradients were consistent across areas concerning the degree of urbanisation and distribution of immigrants (Table II and Supplemental Figure S1). The other SDIs showed weaker explanatory power (*R*^2^ values for the fitted ecological regressions on the spatially smoothed death rates within age groups 60–69 and 70–79, respectively: the four-indicator IMD, 0.113 and 0.127 [Table II]; SDI1, 0.116 and 0.145 [Table II]; SDI2, 0.063 and 0.092; SDI3, 0.084 and 0.076; and SDI4, 0.049 and 0.050).

**Table II.** Associations between SDI1 (i.e. proportion of inhabitants with a low economic standard) and a constructed index of multiple deprivation (four-indicator IMD), on the one hand, and spatially smoothed standardised mortality ratios (SMRs) in year 2019 within the age groups 60–69 and 70–79 years, respectively, on the other hand.

	Age group 60–69 years				Age group 70–79 years			
	Crude death rate per 1000 (no. of deaths)	Relative deviation of average-level SMR from the overall mean (95% CI)^a^	Relative deviation of average-level SMR from the overall mean (95% CI)^a,b^	Relative deviation of average-level SMR from the overall mean (95% CI)^a,c^	Crude death rate per 1000 (no. of deaths)	Relative deviation of average-level SMR from the overall mean (95% CI)^a^	Relative deviation of average-level SMR from the overall mean (95% CI)^a,b^	Relative deviation of average-level SMR from the overall mean (95% CI)^a,c^
*SDI1*:								
Q1	5.1 (1058)	0.94 (0.94–0.95)	0.94 (0.93–0.95)	0.95 (0.94–0.96)	15.5 (2835)	0.92 (0.91–0.93)	0.92 (0.92–0.93)	0.92 (0.91–0.93)
Q2	6.7 (1580)	0.97 (0.96–0.98)	0.97 (0.97–0.98)	0.98 (0.97–0.98)	18.1 (3724)	0.96 (0.96–0.97)	0.96 (0.96–0.97)	0.96 (0.96–0.97)
Q3	7.3 (1702)	– (omitted)	– (omitted)	– (omitted)	19.2 (3981)	– (omitted)	– (omitted)	– (omitted)
Q4	8.2 (1901)	1.02 (1.01–1.03)	1.02 (1.01–1.03)	1.02 (1.01–1.02)	22.4 (4801)	1.04 (1.03–1.04)	1.04 (1.03–1.04)	1.04 (1.03–1.04)
Q5	10.6 (2134)	1.08 (1.07–1.08)	1.07 (1.07–1.08)	1.06 (1.05–1.07)	27.4 (4884)	1.09 (1.09–1.10)	1.10 (1.09–1.10)	1.10 (1.09–1.11)
		*R*^2^=0.116	*R*^2^=0.118	*R*^2^=0.132		*R*^2^=0.145	*R*^2^=0.147	*R*^2^=0.158
*Four-indicator IMD*:								
Q1	5.2 (1078)	0.95 (0.94–0.95)	0.95 (0.94–0.95)	0.95 (0.95–0.96)	15.5 (2840)	0.93 (0.91–0.93)	0.93 (0.92–0.94)	0.92 (0.92–0.93)
Q2	6.4 (1520)	0.97 (0.96–0.97)	0.97 (0.96–0.97)	0.97 (0.96–0.98)	17.9 (3661)	0.96 (0.96–0.97)	0.96 (0.95–0.97)	0.96 (0.95–0.97)
Q3	7.3 (1717)	– (omitted)	– (omitted)	– (omitted)	19.5 (4125)	– (omitted)	– (omitted)	– (omitted)
Q4	8.2 (1881)	1.02 (1.02–1.03)	1.02 (1.02–1.03)	1.02 (1.02–1.03)	22.7 (4829)	1.04 (1.03–1.04)	1.04 (1.03–1.04)	1.04 (1.03–1.05)
Q5	10.9 (2179)	1.07 (1.07–1.08)	1.07 (1.07–1.08)	1.06 (1.05–1.07)	26.9 (4770)	1.08 (1.08–1.09)	1.09 (1.08–1.10)	1.09 (1.08–1.10)
		*R*^2^=0.113	*R*^2^=0.113	*R*^2^=0.129		*R*^2^=0.124	*R*^2^=0.130	*R*^2^=0.141

SDI1 as well as the constructed four-indicator IMD is divided into quintiles, each comprising 1197 small areas (DeSO), where Q1=the least deprived areas and Q5=the most deprived areas.

a Contrast estimates were obtained from an ecological regression of SDI1 or the four-indicator IMD (divided into quintiles) on DeSO-level SMRs (estimated from a Besag, York and Mollie spatial model). Supplemental Figure S1 show the distributions of SMRs within each quintile of SDI1, without and with stratification on grade of urbanisation and distribution of immigrants from non-Western countries, respectively.

b Contrast estimates obtained by adding degree of urbanisation (rural, semi-urban, urban areas) as a covariate.

c Contrast estimates obtained by adding proportion of immigrants from non-Western countries (quintiles) as a covariate.

CI: confidence interval.

## Discussion

Our study suggests that a constructed four-indicator IMD and a single deprivation indicator reflecting economic standard perform essentially equal and outperform the three other single deprivation indicators reflecting educational level, employment status and living in a rented or household-owned apartment/house, respectively, in terms of explanatory power with regard to mortality in the age span 60–79 years. Only four deprivation indicators were compared due to our focus on open-source data.

We validated alternative deprivation measures with regard to mortality only, which poses another limitation of our investigation. Assessing the explanatory power of neighbourhood deprivation on mortality may be relevant for people aged 60–79 but less relevant for young people due to low mortality rates and for elderly people due to subsiding associations between deprivation and death rates (Supplemental Table SI).

## Conclusions

We suggest prioritisation of SDI1, that is, a DeSO-level deprivation indicator based on open-access data on economic standard, for public-health surveillance in Sweden.

## Supplemental Material

sj-docx-1-sjp-10.1177_14034948211030353 – Supplemental material for A comparison of small-area deprivation indicators for public-health surveillance in Sweden Click here for additional data file. Supplemental material, sj-docx-1-sjp-10.1177_14034948211030353 for A comparison of small-area deprivation indicators for public-health surveillance in Sweden by Ulf Strömberg, Amir Baigi, Anders Holmén, Brandon L. Parkes, Carl Bonander and Frédéric B. Piel in Scandinavian Journal of Public Health

sj-docx-2-sjp-10.1177_14034948211030353 – Supplemental material for A comparison of small-area deprivation indicators for public-health surveillance in Sweden Click here for additional data file. Supplemental material, sj-docx-2-sjp-10.1177_14034948211030353 for A comparison of small-area deprivation indicators for public-health surveillance in Sweden by Ulf Strömberg, Amir Baigi, Anders Holmén, Brandon L. Parkes, Carl Bonander and Frédéric B. Piel in Scandinavian Journal of Public Health
