# Supplementary material for: A comparison of small-area deprivation indicators for public-health surveillance in Sweden
Source: Scand J Public Health. 2021 Jul 20;51(4):520–6. doi: 10.1177/14034948211030353 (PMC10259086; doi:10.1177/14034948211030353)
Supplement: sj-docx-1-sjp-10.1177_14034948211030353 – Supplemental material for A comparison of small-area deprivation indicators for public-health surveillance in Sweden [file sj-docx-1-sjp-10.1177_14034948211030353.docx]

**Supplementary Table 1.** Associations between SDI1 (i.e., proportion of inhabitants with low economic standard) and a constructed index of multiple deprivation (4-indicator IMD), on the one hand, and spatially smoothed standardised mortality ratios (SMRs) in year 2019 within the age groups 50-59 and 80-89 years, respectively, on the other hand. SDI1 as well as the constructed 4-indicator IMD is divided into quintiles, each comprising 1,197 small areas (DeSO), where Q1 = the least deprived areas, Q5 = the most deprived areas.

|  | **Age group 50-59 years** | | **Age group 80-89 years** | |
| --- | --- | --- | --- | --- |
|  | **Crude death rate per 1,000**  **(no. of deaths)** | **Relative deviation of average-level SMR from the overall mean (95% CI)^a^** | **Crude death rate per 1,000**  **(no. of deaths)** | **Relative deviation of average-level SMR from the overall mean (95% CI)^a^** |
| ***SDI1:*** | | | | |
| Q1 | 1.7 (482) | 0.969 (0.966-0.972) | 60.5 (3,724) | 0.945 (0.933-0.954) |
| Q2 | 2.2 (584) | 0.990 (0.987-0.993) | 67.0 (5,110) | 0.979 (0.968-0.989) |
| Q3 | 2.5 (643) | - (omitted) | 71.2 (6,294) | - (omitted) |
| Q4 | 3.0 (741) | 1.015 (1.012-1.018) | 73.3 (7,520) | 1.023 (1.013-1.034) |
| Q5 | 3.7 (865) | 1.023 (1.020-1.027) | 79.6 (7,548) | 1.052 (1.041-1.063) |
|  |  | R^2^ = 0.078 |  | R^2^ = 0.032 |
| ***4-indicator IMD:*** | | | | |
| Q1 | 1.7 (469) | 0.971 (0.968-0.974) | 60.9 (3,798) | 0.946 (0.936-0.957) |
| Q2 | 2.2 (603) | 0.991 (0.987-0.994) | 68.0 (5,202) | 0.983 (0.973-0.993) |
| Q3 | 2.4 (625) | - (omitted) | 69.7 (6,068) | - (omitted) |
| Q4 | 2.9 (714) | 1.013 (1.009-1.016) | 75.7 (7,822) | 1.035(1.024-1.045) |
| Q5 | 3.8 (904) | 1.025 (1.021-1.028) | 77.4 (7,306) | 1.038 (1.028-1.049) |
|  |  | R^2^ = 0.071 |  | R^2^ = 0.027 |

**^a^** Contrast estimates were obtained from an ecological regression of SDI1 or the 4-indicator IMD (divided into quintiles) on DeSO-level SMRs (estimated from a Besag, York and Mollie spatial model). The R^2^–values, i.e. the total explained variance by each fitted regression model, are presented in the table.
