# Supplementary material for: A comparison of small-area deprivation indicators for public-health surveillance in Sweden
Source: Scand J Public Health. 2021 Jul 20;51(4):520–6. doi: 10.1177/14034948211030353 (PMC10259086; doi:10.1177/14034948211030353)
Supplement: sj-docx-2-sjp-10.1177_14034948211030353 – Supplemental material for A comparison of small-area deprivation indicators for public-health surveillance in Sweden [file sj-docx-2-sjp-10.1177_14034948211030353.docx]

Supplementary Figure 1

Box-plots of the distributions of SMRs within each quintile of single deprivation indicator 1 (SDI1), i.e. proportion of inhabitants with low economic standard (Q1 = the least deprived, Q5 = the most deprived)

N.B. log-scaled y-axis


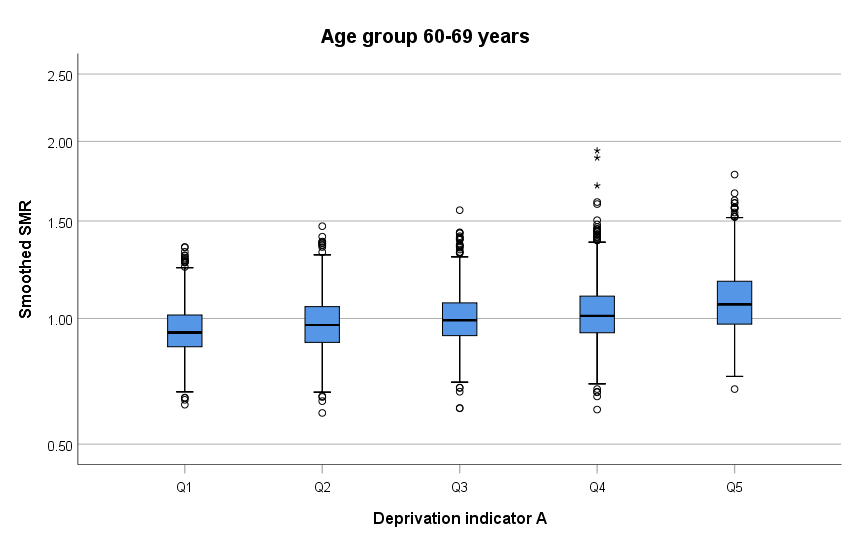


**SDI1**


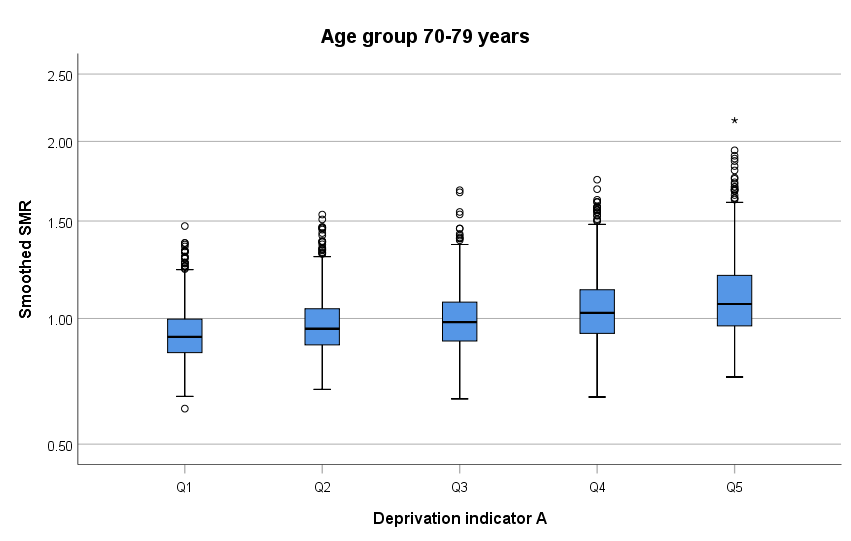


**SDI1**


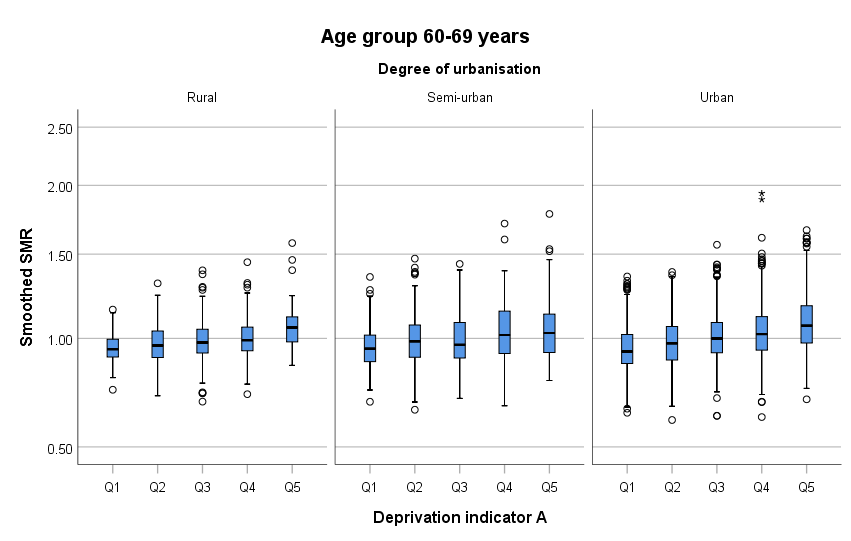


**SDI1**


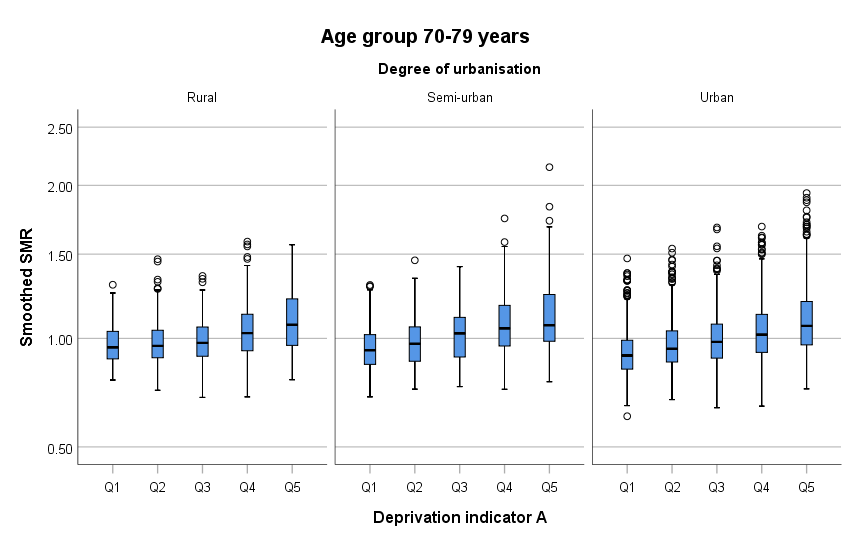


**SDI1**


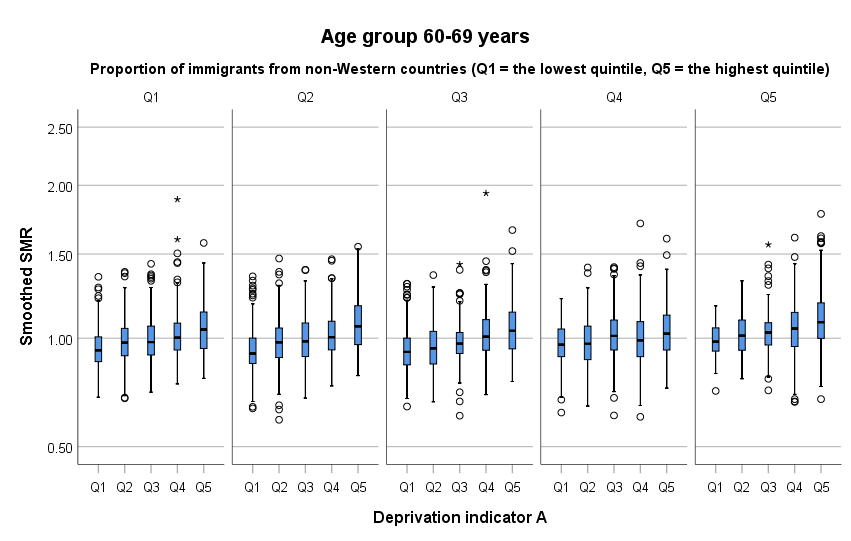


**SDI1**


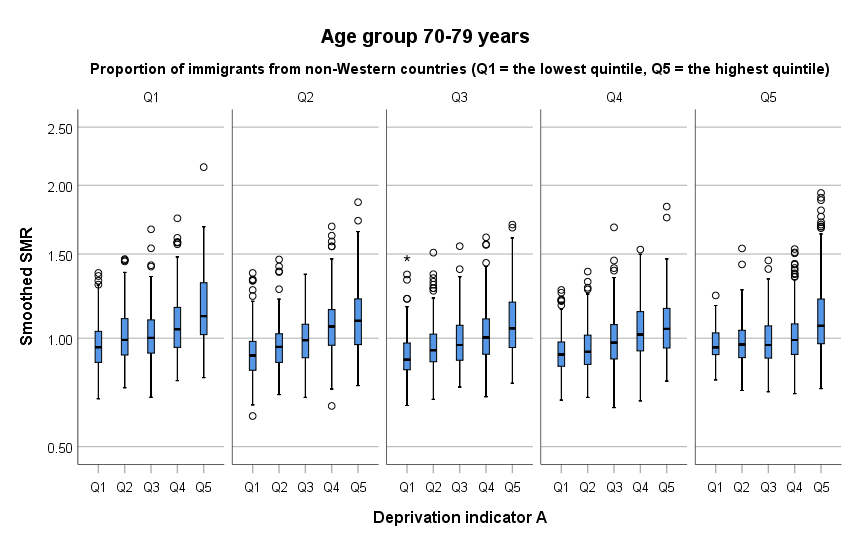


**SDI1**
